# PyBSASeq: a simple and effective algorithm for bulked segregant analysis with whole-genome sequencing data

**DOI:** 10.1186/s12859-020-3435-8

**Published:** 2020-03-06

**Authors:** Jianbo Zhang, Dilip R. Panthee

**Affiliations:** Department of Horticultural Science, North Carolina State University, Mountain Horticultural Crops Research and Extension Center, 455 Research Drive, Mills River, NC 28759 USA

**Keywords:** Bulked segregant analysis, BSA-Seq, PyBSASeq, QTL, SNP-trait association

## Abstract

**Background:**

Bulked segregant analysis (BSA), coupled with next-generation sequencing, allows the rapid identification of both qualitative and quantitative trait loci (QTL), and this technique is referred to as BSA-Seq here. The current SNP index method and G-statistic method for BSA-Seq data analysis require relatively high sequencing coverage to detect significant single nucleotide polymorphism (SNP)-trait associations, which leads to high sequencing cost.

**Results:**

We developed a simple and effective algorithm for BSA-Seq data analysis and implemented it in Python; the program was named PyBSASeq. Using PyBSASeq, the significant SNPs (sSNPs), SNPs likely associated with the trait, were identified via Fisher’s exact test, and then the ratio of the sSNPs to total SNPs in a chromosomal interval was used to detect the genomic regions that condition the trait of interest. The results obtained this way are similar to those generated via the current methods, but with more than five times higher sensitivity. This approach was termed the significant SNP method here.

**Conclusions:**

The significant SNP method allows the detection of SNP-trait associations at much lower sequencing coverage than the current methods, leading to ~ 80% lower sequencing cost and making BSA-Seq more accessible to the research community and more applicable to the species with a large genome.

## Background

Bulked segregant analysis (BSA) has been widely utilized in the rapid identification of trait-associated genetic markers for a few decades [1, 2]. The essential part of a BSA study is to construct two bulks of individuals that have contrasting phenotypes (e.g., tallest plants vs. shortest plants or resistant plants vs. susceptible plants) from segregating populations. If a gene does not contribute to the trait phenotype, its alleles would be randomly segregated in both bulks; whereas if a gene is responsible for the trait phenotype, its alleles would be enriched in either bulk, e.g., one bulk has more allele *A* while the other bulk has more allele *a*. BSA was primarily used to develop genetic markers for detecting gene-trait association at its early stage [1, 2]. The application of next-generation sequencing technology to BSA has eliminated the time-consuming and labor-intensive marker development and genetic mapping steps and has dramatically sped up the detection of gene-trait associations [3–20]. This technique was termed either QTL-seq or BSA-Seq in different publications [5, 6, 21]; we adapted the latter here because it can be applied to study both qualitative and quantitative traits.

The widely used approach in analyzing BSA-Seq data is the SNP index method [5]. For each SNP, the base that is the same as in the reference genome is termed reference base (REF), and the other base is termed alternative base (ALT); the SNP index (allele frequency) of an SNP is calculated by dividing its ALT read with the total read (REF read + ALT read) in a bulk. The greater the Δ(SNP index) (the difference of the SNP indices between bulks), the more likely the SNP contributes to the trait of interest or is linked to a gene that controls the trait. The second approach is the G-statistic method [21]. For each SNP, a G-statistic value is calculated via G-test using the REF read and the ALT read values in each bulk. The SNP with a high G-statistic value would be more likely related to the trait. Both methods identify SNP-trait associations via quantifying the REF/ALT enrichment of a single SNP, and some of the major QTLs can be detected only with high sequencing coverage [3, 5, 22], which leads to high sequencing cost and limits the application of BSA-Seq to the species with a large genome. When this manuscript was in review, a new algorithm termed BRM was published. The authors claimed that the BRM had higher sensitivity than the current methods (10.1093/bioinformatics/btz861).

In BSA studies, bulking enriches the trait-associated alleles in either bulk. The more a gene contributes to the phenotype, the more its alleles are enriched, and so are the SNPs within the gene (one bulk contains more REF read while the other bulk contains more ALT read). The SNPs flanking this gene should be enriched as well due to linkage disequilibrium, the closer the SNP to the gene, the more enrichment is achieved. Such SNPs are termed trait-associated SNPs. Based on the above reasoning, we developed a novel, simple, and effective algorithm for analysis of the BSA-Seq data via quantifying the enrichment of likely trait-associated SNPs in a chromosomal interval. The algorithm was implemented in Python and the script was named PyBSASeq. The sequence data of Yang et al. [3] was used to test our algorithm, and our method detected more QTLs than the current methods [3, 22] even with lower sequencing coverage.

### Implementation

The significant SNP method was implemented in Python, and the code and its detailed usage are available on the website https://github.com/dblhlx/PyBSASeq. The Python implementation of the SNP index method and the G-statistic method can be accessed on https://github.com/dblhlx/. The input file for these scripts are generated via SNP calling, see the Method section for details. The workflow of the scripts is as follows:

1. Read the .tsv input file generated via SNP calling into a Pandas DataFrame;
2. Perform SNP filtering on the Pandas DataFrame;
3. Identify the significant SNPs via Fisher’s exact test (the significant SNP method), calculate the Δ(SNP index) values (the SNP index method), or calculate the G-statistic values (the G-statistic method) using the four allele depth (AD) values (AD_REF1_ and AD_ALT1_ of bulk 1 and AD_REF2_ and AD_ALT2_ of bulk 2) of each SNP in the filtered Pandas DataFrame;
4. Use the sliding window algorithm to plot the sSNP/totalSNP ratios, the Δ(SNP index) values, or the G-statistic values against their genomic positions;
5. Calculate the threshold of the sSNP/totalSNP ratio, the Δ(SNP index), or the G-statistic via simulation. The thresholds were used to identify the significant peaks in the plots generated in step 4.

Two files, PyBSASeq.pdf and BSASeq.csv, will be generated after the successful completion of the PyBSASeq script. PyBSASeq.pdf contains plots with the chromosomal distributions of sSNPs, total SNPs, and sSNP/totalSNP ratios, while BSASeq.csv contains information for all the potential significant peaks including the sliding window-specific thresholds of these peaks.

### SNP filtering

The GATK4-identified SNPs are filtered using the following parameters in order: 1) the unmapped SNPs or SNPs mapped to the mitochondrial or chloroplast genome; 2) the SNPs with an ‘NA’ value in any column of the DataFrame; 3) the SNPs with more than one ALT bases; 4) the SNPs with GQ score less than 20.

### Identification of significant SNPs

The Python module fisher (https://github.com/brentp/fishers_exact_test) or scipy.stats.fisher_exact is used for Fisher’s exact test. The former can take four one-dimensional numpy arrays as input and hence is much faster when dealing with a large dataset. Whereas the latter can only take a numpy array or a Python list ([[AD_REF1_, AD_ALT1_], [AD_REF2_, AD_ALT2_]]) as input. When performing Fisher’s exact test on the real SNP dataset, a SNP with its *p*-value less than 0.01 is defined as a significant SNP.

### Calculation of **Δ**(SNP index) and G-statistic

The Δ(SNP index) of each SNP in the SNP dataset is calculated as below:

$$ \varDelta \left( SNP\  index\right)=\frac{A{D}_{ALT2}}{D{P}_2}-\frac{A{D}_{ALT1}}{D{P}_1} $$

The formula below is used for calculating the G-statistic of each SNP, where *O* is the observed AD (AD_REF1_, AD_ALT1_, AD_REF2_, or AD_ALT2_), *E* is the expected AD under the null hypothesis and is calculated as in the original G-statistic method [21], and *ln* denotes the natural logarithm.

$$ G=2\sum \limits_i{O}_i\times \mathit{\ln}\left({O}_i/{E}_i\right) $$

### Sliding windows

The sliding window algorithm is utilized to aid the visualization (plotting) in BSA-Seq data analysis. The window size is 2 Mb, and the incremental step is 10,000 bp. Most of the sliding windows contain hundreds or thousands of SNPs, and some of them could be significant SNPs. For the significant SNP method, the sSNP/totalSNP ratio of a sliding window is the ratio of the number of sSNPs to the total number of SNPs in the sliding windows. A sliding window containing a trait-controlling gene or with such a gene nearby would have a high sSNP/totalSNP ratio because of phenotypic selection via bulking; the more the gene contributes to the trait, the higher the sSNP/totalSNP ratio. For the SNP index method and the G-statistic method, the Δ(SNP index) or G-statistic of a sliding window is the average values of all the SNPs in the sliding window. A sliding window containing a trait-controlling gene or with such a gene nearby would have a high absolute Δ(SNP index) or G-statistic as well.

Empty windows would be encountered if the amount of SNPs is too low or the SNP distribution is severely skewed. If a sliding window has zero SNP, its sSNP/totalSNP ratio, G-statistic value, or Δ(SNP index) will be replaced with the value of the previous sliding window. If the first sliding window of a chromosome is empty, the string ‘empty’ will be assigned to this sliding window as a placeholder that will be replaced with a non-empty value of the nearest window later.

### Simulation of AD_REF_/AD_ALT_ for threshold calculation

The python module numpy.random.binomial (DP, alleleFreq) is used to calculate the simulated AD_REF_ (smAD_REF_) and simulated AD_ALT_ (smAD_ALT_) of a SNP in a bulk. *DP* is the real depth per sample value of the SNP in the bulk, and *alleleFreq* is the frequency of the ALT base in the bulk under the null hypothesis that the SNP is not associated with the trait. *alleleFreq* is 0.5 in an F_2_ population or 0.75/0.25 in a backcross population, its value in the bulk is obtained via simulation (see the *smAlleleFreq* function of the Python script for details), which should be very close to 0.5 or 0.75/0.25. The module returns the smAD_ALT_, and the smAD_REF_ can be calculated as below:

$$ smA{D}_{REF}= DP- smA{D}_{ALT} $$

### Calculation of the sSNP/totalSNP thresholds

It takes around two minutes to calculate the threshold of a single sliding window via simulation on a relatively powerful desktop computer (Intel Core I7–6700 3.40 Ghz Processor and 32 Gb ram), and calculating a threshold for every sliding window of the SNP dataset via simulation would take a very long time. To overcome this obstacle, we first calculate a genome-wide threshold via resampling, peak sliding windows above this threshold are identified as potential significant peaks, then sliding window-specific thresholds are calculated via simulation to verify if the sSNP/totalSNP ratios of these peak sliding windows are really significant.

#### Genome-wide threshold

The amount of SNPs that are the same as the average number of SNPs per sliding window are randomly selected from the entire SNP dataset. For each SNP in this sample, smAD_REF1_/smAD_ALT1_ of bulk 1 and smAD_REF2_/smAD_ALT2_ of bulk 2 are obtained via simulation. These simulated AD values of both bulks are used to perform Fisher’s exact test. A SNP with its *p*-value less than 0.10 is considered an sSNP. The sSNP/totalSNP ratio of this sample is calculated and recorded. This process is repeated 10,000 times, and the 99.5th percentile of these 10,000 simulated sSNP/totalSNP ratios is used as the significant threshold for the detection of potential significant peaks. A higher cut-off p-value (0.01 is used in the real SNP dataset) is used here, resulting in the identification of more significant SNPs from the simulated SNP sub-dataset, hence a higher threshold and less false positives.

#### Sliding window threshold

For each SNP in a sliding window, smAD_REF1_/smAD_ALT1_ of bulk 1 and smAD_REF2_/smAD_ALT2_ of bulk 2 are obtained via simulation, and Fisher’s exact test, identification of significant SNPs, and sSNP/totalSNP calculation are performed in the same way as above. This process is repeated 10,000 times, and again the 99.5th of these 10,000 simulated sSNP/totalSNP ratios is used as the threshold for this sliding window.

### Calculation of the **Δ**(SNP index) and G-statistic thresholds

For each SNP in the SNP dataset, smAD_REF1_/smAD_ALT1_ of bulk 1 and smAD_REF2_/smAD_ALT2_ of bulk 2 are obtained via simulation. Using these AD values, the Δ(SNP index) or the G-statistic of each SNP is calculated as above . This process is repeated 10,000 times, the 99% confidence interval of the 10,000 Δ(SNP index) values is used as a significant threshold for the SNP index method, and the 99.5th percentile of the 10,000 G-statistic values is used as a significant threshold for the G-statistic method. Please note that the threshold of the Δ(SNP index) or the G-statistic is at the SNP level while the threshold of the sSNP/totalSNP ratio is at the sliding window level.

## Results

### Identification of significant SNPs

In BSA-Seq studies, if a SNP is not associated with the trait, its REF/ALT reads would be randomly segregated in both bulks, and the ALT (or REF) read proportions in two bulks should be similar; however, if a SNP is associated with the trait, its REF/ALT reads would be enriched in either bulk due to phenotypic selection via bulking, and the ALT (or REF) read proportions should be significantly different between the bulks. Fisher’s exact test, G-test, or chi-square test can be used to identify such trait-associated SNPs from the SNP dataset, but Fisher’s exact test is more accurate when the sample size is small. For the same set of 2 × 2 contingency table, the *p*-value calculated via either G-test or chi-square test is less than that calculated via Fisher’s exact test, even for sample sizes in the hundreds. To decrease the chance of false positives, Fisher’s exact test was used to identify the likely trait-associated SNPs here, as did by many others [4, 20]. A small *p*-value of the Fisher’s exact test suggests that the ALT proportion difference of a SNP between bulks is more likely caused by bulking, and an SNP with its p-value less than 0.01 was considered more likely associated with the trait and was termed significant SNP (sSNP) here. 240,351 sSNPs were identified among total 1,303,084 filtered SNPs (see the Implementation section for the filter criteria), and the chromosomal distribution of SNPs was summarized in Table 1. The chromosomes 8, 1, 2, 10, and 5 contained the most sSNPs and had the highest sSNP/totalSNP ratios, correlating perfectly with the chromosomes carrying the verified QTLs [3, 22].

**Table 1:** Chromosomal distribution of SNPs

Chromosome	sSNP	totalSNP	sSNP/totalSNP
1	52,093	160,780	0.324
2	48,912	125,059	0.391
3	3502	45,927	0.076
4	3743	62,317	0.060
5	15,482	102,474	0.151
6	7653	159,857	0.048
7	12,679	128,658	0.099
8	54,372	132,646	0.410
9	1709	57,971	0.029
10	28,711	98,646	0.291
11	5235	180,319	0.029
12	6260	48,430	0.129
Genome-wide	240,351	1,303,084	0.184

### Enrichment of sSNPs

**Fig. 1:**
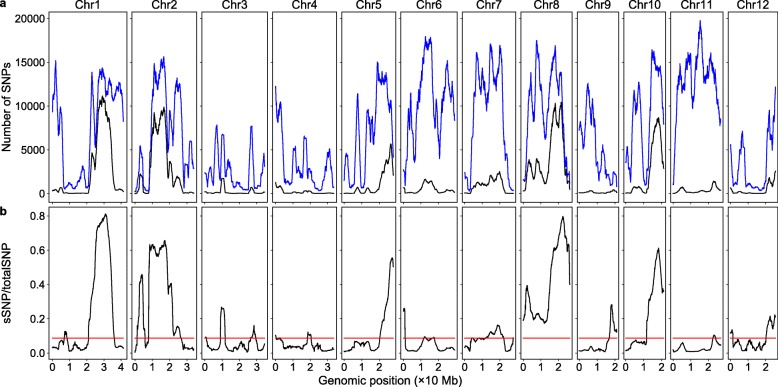
Genomic distributions of SNPs and sSNPs/totalSNP ratios. The red horizontal lines are the thresholds obtained via resampling. **a** The sSNPs (black) and total SNPs (blue). **b** The ratio of sSNPs to total SNPs

The sSNPs should cluster around the genes controlling the trait phenotype on the chromosomes due to linkage disequilibrium. Using the sliding window technique, the number of sSNPs was plotted across all the chromosomes to test if this was the case. We found the sSNP plot approximately matched with the major peaks in plots produced by the SNP index method and the G-statistic method [3, 22] (Fig. 1a). However, counting the absolute number of sSNPs is not an ideal way to measure the sSNP enrichment because SNPs were distributed unevenly across and between chromosomes (Fig. 1a); if a gene that conditions the trait is located in a region with fewer SNPs, it would be missed using this approach. Thus, we used the ratio of sSNPs to total SNPs in a chromosomal region to measure the sSNP enrichment. The sSNP/totalSNP ratios were plotted for all the chromosomes (Fig. 1b), and the plot pattern matched very well with that produced by the G-statistic method [3, 22]. The most obvious difference between Fig. 1a and b was the first peak on chromosome 2 and the peaks on chromosomes 3, 6 and 9; these regions contained fewer SNPs, but the sSNPs enrichment was relatively high.

As stated in the Implementation section, calculating the sSNP/totalSNP ratio threshold of a sliding window via simulation takes around two minutes, and the entire SNP dataset contained 34,919 sliding windows. Calculation of the thresholds of all the sliding windows would take more than a month, thus resampling was utilized to obtain a genome-wide threshold (see the Implementation section) to identify potentially significant peaks in Fig. 1b. The threshold obtained this way was 0.087. In addition to the six major QTLs (two of them on chromosome 2) verified in the work of Yang et al. [3], one or more new peaks on all chromosomes except chromosomes 5 and 10 were also above the threshold (Fig. 1b).

The genome-wide threshold was acquired using the sample size equal to the average number of SNPs of the sliding windows. We tried different sample sizes for the genome-wide threshold calculation, and the results demonstrated that increasing the sample size decreased the threshold (not shown here). The number of SNPs in sliding windows varied drastically across the genome (Fig. 1a), hence the thresholds should vary between sliding windows, and some of the significant peaks in Fig. 1b could be false positives if they contained a low number of SNPs. Thus, we calculated sliding window-specific thresholds for all the potential significant peaks in Fig. 1b via simulation. The results revealed that most of the sliding window thresholds were very close to the genome-wide threshold, except the sliding windows with a very low number of SNPs. Using the sliding windows thresholds, only the first peak on chromosome 3 was identified as a false positive; this peak sliding window had only 2260 SNPs, less than 1/3 of the average number of SNPs per sliding window (6984), and its sSNP/totalSNP ratio was 0.0929, very closed to the genome-wide threshold (Table S1). Since the sliding windows with a higher number of SNPs tends to have a lower threshold, the peaks with their sSNP/totalSNP ratios lower than the genome-wide threshold and containing a very high number of SNPs might be false negatives using above approach; however, these genomic regions should have very small phenotypic effects judged by their low sSNP/totalSNP ratios.

### Sequencing coverage affected the detection of SNP-trait association

**Fig. 2:**
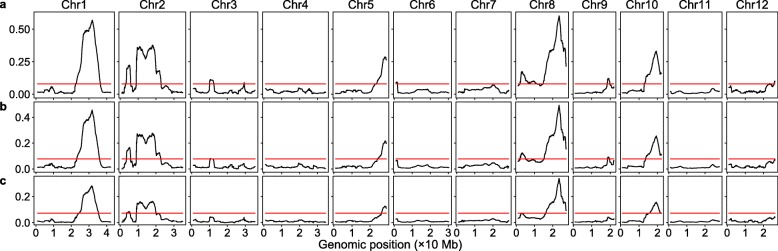
Genomic distribution of sSNP/totalSNP ratios at different sequencing coverage levels. The red horizontal lines are the thresholds obtained via resampling. **a** 40% of the original sequence reads. **b** 30% of the original sequence reads. **c** 20% of the original sequence reads

Using the Lander/Waterman equation [23], the sequencing coverage of SRR834927 and SRR834931 was estimated to be 84× and 103×, respectively. It would be very costly to achieve such high sequencing coverage for the organisms with a large genome. Thus, we wanted to know how decreasing sequencing coverage would affect the detection of SNP-trait associations. To achieve lower sequencing coverage, we sampled 40%, 30%, and 20% of the raw sequence reads using the seqtk program (https://github.com/lh3/seqtk) with different random seeds. Random seeds were used here just to ensure that paired sequences in the same bulk were selected when sampling. The sSNPs were identified from these sequence subsets and the ratios of sSNP/totalSNP were plotted along all the chromosomes as above. The results revealed that the plotting patterns were very similar at different sequencing coverage levels (Fig. 2); with decreasing sequencing coverage, the total SNPs decreased slightly, while the number of sSNP and the sSNP/totalSNP ratio decreased substantially (Table S2). Because the threshold did not change as much, more and more minor SNP-trait associations were missed with decreasing sequencing coverage. However, with 40%, 30%, or even 20% of the original sequencing coverage, more QTLs were detected than the current methods with the original sequencing coverage [3, 22].

We calculated the sliding window-specific thresholds for all the potential significant peaks in Fig. 2 as well, and only one peak on chromosome 9 at 30% of the original sequencing coverage was identified as a false positive (Fig. 2b and Table S1), again, this peak contained a very low amount of SNPs (963). All the seven peaks identified in Fig. 2c were still significant using the sliding window-specific thresholds (Table S1). Although not obvious in Figs. 1b and 2, positions of many significant peaks were not the same at different sequencing coverage levels, the difference was very minor for all but one peak on chromosome 2, which shifted 1.86 Mb at 30% of the original sequencing coverage (Table S1). This peak was very close to the centromere [3], and the recombination frequency around this region should be low. The curve around this peak was very noise in Fig. 1b; it was not surprising that down-sampling led to significant peak shifting.

### Sensitivity comparison

The results in Figs. 1b and 2 indicated that the significant SNP method had higher detection power. However, different methods were used to generate the SNP datasets [3, 22], which might lead to different detection sensitivities. To rule out this possibility, we implemented the SNP index method and the G-statistic method in Python and tested all the three methods with the same SNP dataset. First, we tested if the results of Yang et al. and Mansfeld and Grumet can be replicated using our scripts. As in the studies mentioned above, the SNP dataset was filtered with the following criteria: fb.GQ ≥ 99, sb.GQ ≥ 99, fb.DP ≥ 40, sb.DP ≥ 40, fb.DP + sb.DP ≥ 100, and fb.DP + sb.DP ≤ 400. Although the SNP datasets were generated in different ways (GATK4 vs. GATK vs. Samtools) and no smoothing besides the sliding window algorithm was applied in our scripts, the results, including the plot patterns, the G-statistic values, and the Δ(SNP index) values and its confidence intervals, were very similar [3, 22], and the positions of the peaks/valleys matched almost perfectly between different approaches (Figure S1). A non-parametric method was used to calculate the threshold in the G-statistic method by Yang et al. and Mansfeld and Grumet, and different approaches were used to remove the G-statistic values from the QTL regions. Thus, the thresholds were a little different in these studies, and so were the QTL detection results [3, 22]. In our G-statistic script, we used simulation for threshold calculation (see the Implementation section), and the thresholds obtained this way were consistent across all the chromosomes and was less conservative than the previously reported approaches. Using the high sequencing depth SNP subset, similar results were obtained by both the SNP index method and the G-statistic method: the six major QTLs and a minor QTL on chromosome 2 were detected (Figure S1). However, the significant SNP method had the highest sensitivity using the same filtering criteria, and it can detect more minor QTLs than other methods even if the whole SNP dataset was used (Figs. 1b, 2, and Figure S1). Please note that 99% confidence interval was used for the calculation of the threshold in the SNP index method, and the 99.5th percentile was used for the calculation of the threshold in the G-statistic method or the significant SNP method.

**Fig. 3:**
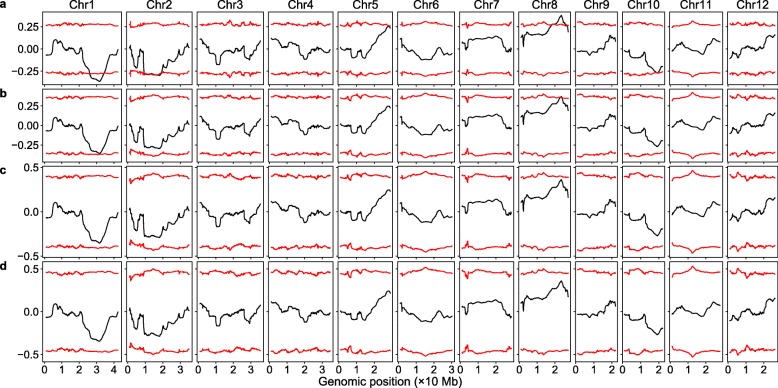
Genomic distribution of ∆(SNP index) at different sequencing coverage levels. The red curves indicate 99% confidence intervals obtained via simulation. **a** The original sequence reads. **b** 40% of the original sequence reads. **c** 30% of the original sequence reads. **d** 20% of the original sequence reads

**Fig. 4:**
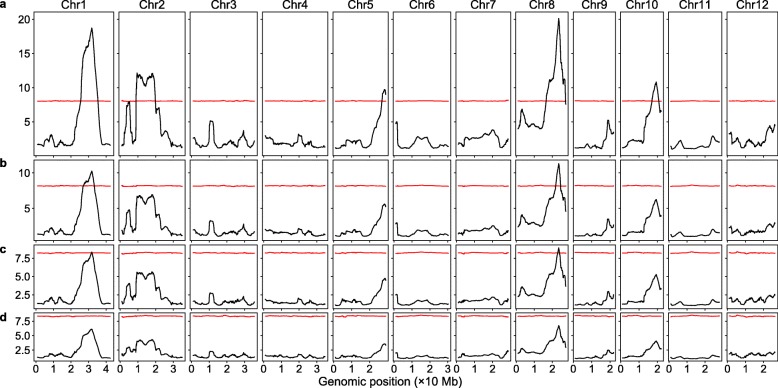
Genomic distribution of G-statistic at different sequencing coverage levels. The red curves are the G-statistic thresholds obtained via simulation. **a** The original sequence reads. **b** 40% of the original sequence reads. **c** 30% of the original sequence reads. **d** 20% of the original sequence reads

As in the significant SNP method, we also tested how decreasing sequencing coverage would affect the detection of the SNP-trait associations in these two methods. Using the original sequencing reads, the SNP index method had relatively low detection power, the major QTL on chromosome 5 was missed and the peak (valley) representing the major QTL on chromosome 10 was barely beyond the threshold. With decreasing sequencing coverage, the Δ(SNP index) did not change much, but the thresholds increased dramatically, the QTLs on chromosomes 2, 5, and 10 were missed at 40% of the original sequencing coverage and all the QTL were missed at 30% or lower of the original sequencing coverage (Fig. 3). For the G-statistic method, with the original sequencing reads, all the 6 major QTLs can be detected. With decreasing sequencing coverage, the G-statistic values decreased substantially, whereas the threshold increased slightly; the QTLs on chromosomes 2, 5, and 10 were missed at 40% of the original sequencing coverage, the peaks representing the QTLs on chromosomes 1 and 8 were barely above the threshold at 30% of the original sequencing coverage, and all the QTLs were missed at 20% of the original sequencing coverage (Fig. 4).

## Discussion

The significant SNP method detected more than 10 minor QTLs along with all of the major QTLs detected via the current methods when run with the entire SNP dataset based on the original sequencing reads (Figs. 1b, 3a, and 4a). Plant cold tolerance is a complex quantitative trait controlled by many genes [24, 25]. The additional QTLs detected via the significant SNP method may represent the minor QTLs that have small phenotypic effects. Filtering out the SNPs with a low DP value increased the sensitivity of the current methods (Figure S1, Figs. 3 and 4), but doing so increased the sensitivity of the significant SNP method as well (Figure S1b and Fig. 1b). Decreasing the sequencing coverage substantially reduced the detection power of all the methods (Figs. 2, 3, and 4). At 20% of the original coverage (17× in the first bulk and 21× in the second bulk), all QTLs were missed using the current methods; however, all the verified major QTLs plus one minor QTL can still be detected via the significant SNP method, manifesting that the significant SNP method is at least five times more sensitive.

Because of its high sensitivity, the intervals of the QTLs (chromosomal regions above the threshold) were quite wide (Fig. 1b). An extreme case was chromosome 8 where all of its sSNP/totalSNP ratios were greater than the threshold, which does not imply that all the SNPs on chromosome 8 were involved in conditioning the cold tolerance trait. The SNPs in the causal locus are enriched because of phenotypic selection via bulking while the SNPs flanking the causal locus are enriched because of linkage disequilibrium. Any recombination event between the SNPs that affect the trait of interest and the SNPs flanking the causal gene would reduce the enrichment of the flanking sSNPs, thus SNPs in the causal locus should have the highest enrichment and should be located in the peak region. Therefore, there are only two QTLs on chromosome 8: a minor one on the proximal arm while a major one on the distal arm of the chromosome. All three methods use the sliding window algorithm to detect the SNP-trait associations and should have the same level of resolution if the sliding window settings (window size and incremental step) are the same.

The major difference between the significant SNP method and the current methods is how the SNP-trait associations are identified. Both the SNP index method and the G-statistic method use SNP-level thresholds to identify significant sliding windows; whereas the significant SNP method uses sliding window-level thresholds to identify significant sliding windows. The average number of SNPs was 6984 in the sliding windows, much higher than the average sequencing coverage in either bulk (84× in the first bulk and 103× in the second bulk), which could be why the significant SNP method has much higher statistical power and is more sensitive in the detection of SNP-trait associations. GATK is widely used for SNP and small InDel calling, and the new version of GATK4 is also capable of copy number and structural variant calling. PyBSASeq is designed to analyze the GATK-generated variant calling data, though it has only been tested for analysis of the SNP and small InDel calling data, it should be able to handle the GATK4-generated copy number variant and structural variant data as well.

## Conclusions

The high sensitivity of the significant SNP method allows the detection of SNP-trait associations at reduced sequencing coverage, leading to reduced sequencing costs. Thus, BSA-Seq can be more practically applied to species with a large genome.

## Methods

The sequencing data used in this study were generated by Yang et al. [3]. Using the G-statistic method, Yang et al. identified six major cold tolerance QTLs in rice and five of them were consistent with the then available QTL database or previous publications. The *Oryza sativa subsp. japonica* rice cultivar Nipponbare was used as one of the parents in generating the F_3_ population of the BSA-Seq experiment, and its genome sequence was used as the reference sequence for SNP calling in our study. The size of the F_3_ population was 10,800 (plants), and the extremely cold-sensitive bulk (ES) contained 430 plants while the extremely cold-tolerant bulk (ET) contained 385 plants. The bulked DNA libraries were sequenced using the Illumina Hiseq 2000 sequencing platform, ~ 360 million 101 bp pair-end reads were obtained from the ES bulk and ~ 440 million 101 bp pair-end reads were obtained from the ET bulk [3].

### Sequencing data preprocess

The raw sequences (SRR834927 and SRR834931) for BSA-Seq analysis are downloaded from NCBI using fasterq-dump (https://github.com/ncbi/sra-tools). Quality control, adapter trimming, quality filtering, per-read quality pruning of the downloaded sequences are performed using fastp at the default setting [26].

### SNP calling

The preprocessed sequences are aligned to the ‘Nipponbare’ reference genome sequence (Release 41, downloaded from https://plants.ensembl.org/Oryza_sativa/Info/Index) using BWA [27–29]. SNP calling is carried out following the best practice of Genome Analysis Toolkit (GATK) [30] and the Genome Analysis Toolkit 4 (GATK4) tool documentation on the GATK website https://software.broadinstitute.org/gatk/documentation/tooldocs/current/. The GATK4-generated .vcf file usually contains the information for two bulks, which are termed the first bulk (fb) and the second bulk (sb), respectively. Using the GATK4 tool, a .tsv (tab-separated value) file is generated using the relevant columns (CHROM, POS, REF, ALT, fb.AD, fb.GQ, sb.AD, sb.GQ) of this .vcf file; Table 2 shows the first five rows of this .tsv file.

**Table 2:** The first five rows of the GATK4 output file

CHROM^a^	POS^b^	REF^c^	ALT^d^	834927.AD^e^	834927.GQ^f^	834931.AD^e^	834931.GQ^f^
1	29,759	C	G	0,2	6	0,2	6
1	31,071	A	G	25,39	99	33,29	99
1	31,478	C	T	27,38	99	48,32	99
1	33,667	A	G	21,46	99	39,32	99
1	34,057	C	T	29,37	99	32,31	99

^a^The chromosome on which the SNP is located

^b^The position of the SNP on the chromosome

^c^The base sequence of the SNP that is the same as the one from the reference genome

^d^The base sequence that is different from REF

^e^The allele depths (AD) of the SNP in the first bulk (ID: 834927) or the second bulk (ID: 834931). This column contains two numbers, the first one is the REF read (AD_REF_) and the second is the ALT read (AD_ALT_)

^f^The genotype quality of the SNP in the first bulk (ID: 834927) or the second bulk (ID: 834931)

The number of REF/ALT reads of a SNP is defined as allele depth (AD) in GATK4. Here they are represented as AD_REF_ and AD_ALT_, respectively, and a ‘1’ or ‘2’ is added to its subscript when appropriate to indicate which bulk it belongs to; the same can be applied to the sequencing depth as well. In some rare occasions, the GATK4-generated depth per sample (DP) of an SNP can be either greater or less than the sum of the ADs in a bulk, here the DP of an SNP in a bulk is defined as below for all the SNPs:

$$ DP=A{D}_{REF}+A{D}_{ALT} $$

## Supplementary information

**Additional file 1:** **Table S1.** Verification of potential significant peaks.

**Additional file 2:** **Table S2.** Chromosomal distribution of SNPs at different sequencing coverage levels.

**Additional file 3:** **Figure S1.** Replication of the SNP index method and the G-statistic method in Python.

## Data Availability

The sequences (SRR834927 and SRR834931) used in this study can be downloaded from the NCBI website.

## Abbreviations

AD: Allele depth
ALT: Alternative base
BSA: Bulked segregant analysis
DP: Depth per sample
GATK: Genome Analysis Toolkit
InDel: Insertion or deletion
QTL: Quantitative trait locus
REF: Reference base
SNP: Single nucleotide polymorphism
sSNP: Significant SNP
