# Supplementary material for: PyBSASeq: a simple and effective algorithm for bulked segregant analysis with whole-genome sequencing data
Source: BMC Bioinformatics. 2020 Mar 6;21:99. doi: 10.1186/s12859-020-3435-8 (PMC7060572; doi:10.1186/s12859-020-3435-8)
Supplement: Supplementary file 2 — Additional file 2: Table S2. Chromosomal distribution of SNPs at different sequencing coverage levels. [file 12859_2020_3435_MOESM2_ESM.pdf]

Table S1. Chromosomal distribution of SNPs at different sequencing coverage levels

Chromosome	40% of the original coverage			30% of the original coverage			20% of the original coverage		
	ltaSNP	totalSNP	ltaSNP/totalSNP	ltaSNP	totalSNP	ltaSNP/totalSNP	ltaSNP	totalSNP	ltaSNP/totalSNP
1	28 501	150 662	0.189	20 464	144 815	0.141	11 122	133 953	0.083
2	23 760	116 704	0.204	16 531	111 944	0.148	8727	103 470	0.084
3	1492	43 650	0.034	1030	42 307	0.024	592	39 406	0.015
4	1628	59 037	0.028	1127	56 702	0.020	674	52 609	0.013
5	6865	96 583	0.071	4892	93 201	0.052	2776	86 864	0.032
6	3302	150 246	0.022	2404	145 069	0.017	1361	134 562	0.010
7	5122	120 045	0.043	3549	115 430	0.031	1925	106 121	0.018
8	26 933	123 411	0.218	19 378	118 699	0.163	10 652	108 581	0.098
9	825	55 088	0.015	680	53 291	0.013	424	49 768	0.009
10	13 039	92 815	0.140	9477	89 639	0.106	5526	82 996	0.067
11	2622	171 170	0.015	1963	165 570	0.012	1313	154 588	0.008
12	2589	46 061	0.056	1775	44 596	0.040	995	41 051	0.024
Genome-wide	116 678	1 225 472	0.095	83 270	1 181 263	0.070	46 087	1 093 969	0.042
