# Supplementary figures and images for: PyBSASeq: a simple and effective algorithm for bulked segregant analysis with whole-genome sequencing data
Source: BMC Bioinformatics. 2020 Mar 6;21:99. doi: 10.1186/s12859-020-3435-8 (PMC7060572; doi:10.1186/s12859-020-3435-8)

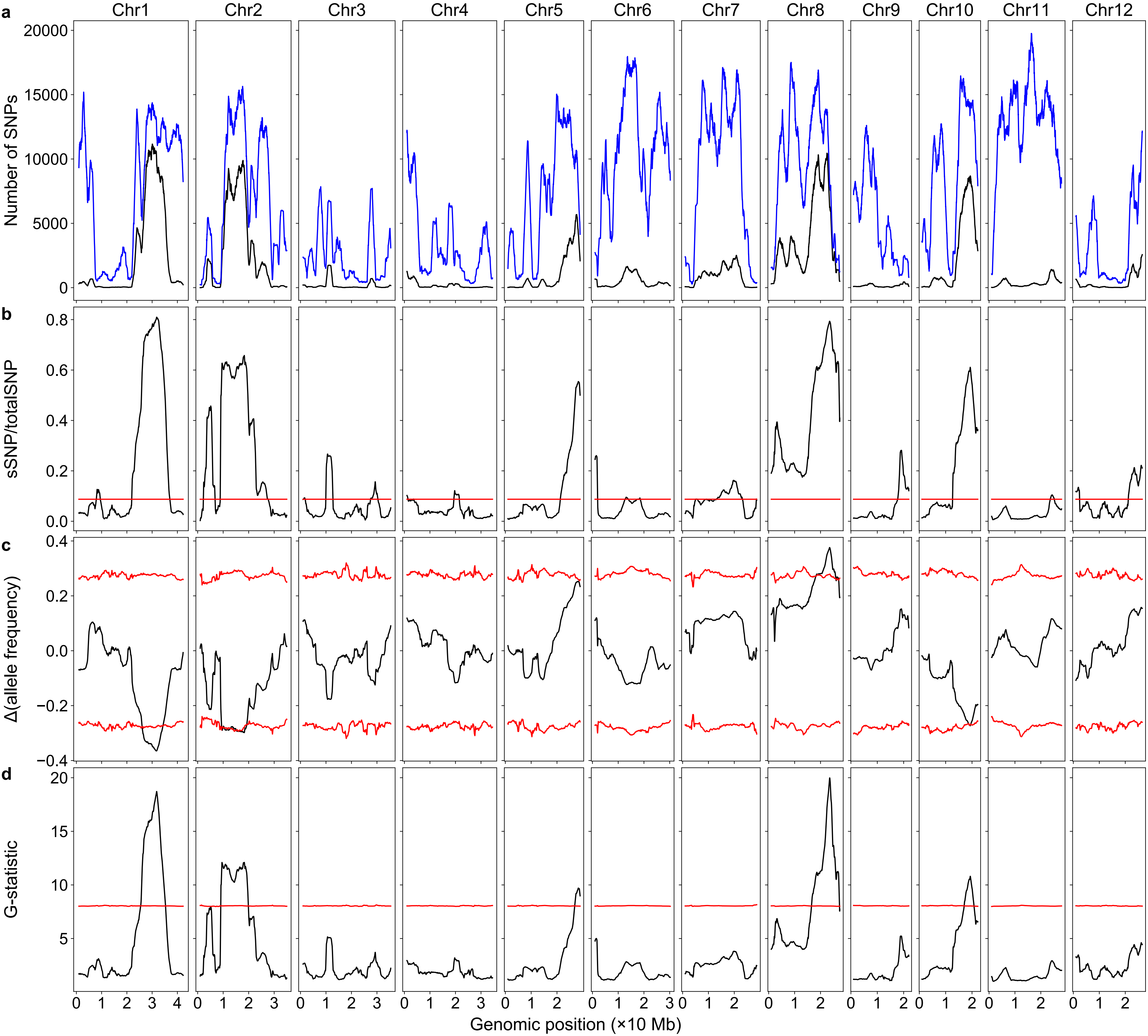

Supplement: Supplementary file 3 — Additional file 3: Figure S1. Replication of the SNP index method and the G-statistic method in Python. [file 12859_2020_3435_MOESM3_ESM.pdf]
